# A robust mRNA signature obtained via recursive ensemble feature selection predicts the responsiveness of omalizumab in moderate‐to‐severe asthma

**DOI:** 10.1002/clt2.12306

**Published:** 2023-11-17

**Authors:** Sarah Kidwai, Pietro Barbiero, Irma Meijerman, Alberto Tonda, Paula Perez‐Pardo, Pietro Lio ´, Anke H. van der Maitland‐Zee, Daniel L. Oberski, Aletta D. Kraneveld, Alejandro Lopez‐Rincon

**Affiliations:** ^1^ Division of Pharmacology Utrecht Institute for Pharmaceutical Science Faculty of Science Utrecht University Utrecht The Netherlands; ^2^ Department of Computer Science and Technology University of Cambridge Cambridge UK; ^3^ UMR 518 MIA INRAE Universite Paris‐Saclay Paris France; ^4^ Department of Pulmonary Medicine Amsterdam UMC, University of Amsterdam Amsterdam The Netherlands; ^5^ Department of Data Science University Medical Center Utrecht Utrecht The Netherlands

**Keywords:** anti‐IgE, asthma, biomarker, machine‐learning, omalizumab

## Abstract

**Background:**

Not being well controlled by therapy with inhaled corticosteroids and long‐acting β2 agonist bronchodilators is a major concern for severe‐asthma patients. The current treatment option for these patients is the use of biologicals such as anti‐IgE treatment, omalizumab, as an add‐on therapy. Despite the accepted use of omalizumab, patients do not always benefit from it. Therefore, there is a need to identify reliable biomarkers as predictors of omalizumab response.

**Methods:**

Two novel computational algorithms, machine‐learning based Recursive Ensemble Feature Selection (REFS) and rule‐based algorithm Logic Explainable Networks (LEN), were used on open accessible mRNA expression data from moderate‐to‐severe asthma patients to identify genes as predictors of omalizumab response.

**Results:**

With REFS, the number of features was reduced from 28,402 genes to 5 genes while obtaining a cross‐validated accuracy of 0.975. The 5 responsiveness predictive genes encode the following proteins: Coiled‐coil domain‐ containing protein 113 (*CCDC113*), Solute Carrier Family 26 Member 8 (*SLC26A*), Protein Phosphatase 1 Regulatory Subunit 3D (*PPP1R3D*), C‐Type lectin Domain Family 4 member C (*CLEC4C*) and *LOC100131780* (not annotated). The LEN algorithm found 4 identical genes with REFS: *CCDC113, SLC26A8 PPP1R3D* and *LOC100131780*. Literature research showed that the 4 identified responsiveness predicting genes are associated with mucosal immunity, cell metabolism, and airway remodeling.

**Conclusion and clinical relevance:**

Both computational methods show 4 identical genes as predictors of omalizumab response in moderate‐to‐severe asthma patients. The obtained high accuracy indicates that our approach has potential in clinical settings. Future studies in relevant cohort data should validate our computational approach.

## 1 INTRODUCTION

Asthma is a common chronic respiratory disease, affecting more than 300 million individuals worldwide.^1^ Around 50%–70% of the total asthmatic population represents individuals with moderate‐to‐severe asthma.^2^, ^3^ This form of asthma is difficult to treat, resulting in frequent asthma exacerbations, hospitalizations, and even death.^4^, ^5^ Patients diagnosed with moderate‐to‐severe asthma cannot always control their disease with long acting inhaled *β* 2‐agonists and corticosteroids.^6^ As an add‐on therapy for these patients, a biological such as omalizumab^7^ can be prescribed. This anti‐IgE agent specifically binds free IgE and prevents IgE binding to the high affinity IgE receptor FcεRI expressed on immune cells such as mast cells, basophils and antigen‐presenting cells.^8^ It reduces allergic airway inflammation by decreasing IgE levels in blood, and by consequent downregulation of the expression of FcεRI on airway inflammatory cells and lowers eosinophil counts.^9^, ^10^, ^11^

Despite omalizumab's widely accepted use, moderate‐to‐severe asthma patients do not always benefit from this anti‐IgE therapy. Because there are also other biologicals such as anti‐IL‐5 (mepolizumab and reslizumab),^12^ anti‐IL‐5R (benralizumab)^13^ and anti‐IL‐4R (dupilimab)^14^ available prediction of treatment response will support choosing the best biological for the individual patient. This will reduce the burden of disease and health care costs that arise due to the use of improper or suboptimal medications. However, predictors of response to omalizumab are still limited.^15^, ^16^, ^17^ Therefore, it is of clinical significance to identify reliable biomarkers as predictors of omalizumab response that can match individual moderate‐to‐severe asthma patients with the most effective medication.

Finding accurate biomarkers for omalizumab response is difficult as these biomarker studies are diverse and complex. Various studies have explored potential biomarkers to predict omalizumab responsiveness, such as genes^18^ or clinical measurements (e.g., total serum IgE, FEV1).^19^, ^20^, ^21^ But the available biomarkers are limited in number and accuracy. Consequently, there is no consensus on the best set of biomarkers of omalizumab therapy for those who do benefit from the drug.

Machine learning (ML), a branch of artificial intelligence, provides a set of techniques that can potentially deal with complex omics data to find the right biomarkers. Typically, omics datasets contain thousands of features (such as genetic variations, mRNA expression, miRNAs expression or metabolites concentrations) in a relatively small number of samples, making it difficult to analyse using standard univariate techniques.^22^, ^23^

Using ML gene cluster analysis, Upchurch et al. (2020)^6^ identified transcriptional differences between omalizumab responders and non‐responders. The study showed 3867 genes which were differentially expressed between healthy controls, responders, and non‐responders. However, this set of response‐predictive genes is too large to be effectively used in a clinical setting. With Recursive Ensemble Feature Selection (REFS)͵^22^ a more accurate and robust gene signature can be found over the gene cluster analysis method that was used by Upchurch. Feature selection (REFS) method shows unbiased gene interactions in contrast to clustering of over/under expressed genes dependent on annotated pathway functions or a single algorithm.^24^, ^25^ REFS allows the identification of compact gene signatures. Logic explained networks (LEN), an advanced computational algorithm, gives in contrast to other algorithms^26^ an interpretable result by providing an explanation for the possible relationships between the features. It simplifies how to read the mRNA expression as a set of logical rules which may serve to take clinical action/decision. Measuring mRNA expression in patients' whole blood could provide a direction for omalizumab therapy responsiveness based on LEN rules.

In this study, we use two ML techniques, REFS and LEN, to reduce the number of features to a more compact size, which is effective for use in a clinical setting.^24^, ^25^

We implemented the two‐state‐of‐art computational algorithm in ML that is, the REFS algorithm and LEN algorithm, on mRNA expression profiles in whole blood of moderate‐to‐severe asthma patients. We used the same samples as those used by Upchurch et al. (2020)^6^ to find predictors of omalizumab response. Single Response‐predictive genes were identified through ML‐based REFS and validated with 10‐fold cross‐validation.^24^, ^25^ In addition, we used LEN^26^ which identifies gene groups and their interrelationship as predictors of omalizumab response. The LEN algorithm provides rules on sets of mRNA expression compared with healthy controls. The biological context and pathway annotation of REFS and LEN overlapping omalizumab response‐predictive genes were validated through literature research to gain a better understanding of mechanisms involved in omalizumab responsiveness.

## 2 METHODS

### 2.1 Patient samples and data

Data on individual moderate‐to‐severe adult asthma patients, whole blood mRNA profiles and clinical information was retrieved from the publicly available database ‘Gene Expression Omnibus’ (GEO) with accession code GSE134544. This dataset was published by Upchurch et al. (2020).^6^

The GSE134544 database has 239 blood mRNA samples from 40 moderate‐to‐severe adult asthmatic patients, and 17 non‐asthmatic healthy controls. Of the 40 patients, 30 were defined as responders and 10 as non‐responders. For the LEN analysis, besides the whole blood mRNA expression of patients, 17 healthy controls were also included. Patients were prescribed omalizumab (Xolair®, Genentech) and were dosed as per the manufacturer's dosing table (according to serum IgE and body weight).

The blood transcriptome included all blood cell types. For every patient, the sample contained 28,402 gene expression levels. Whole blood mRNA expression profiles measured 1 week before the start of treatment of the 40 moderate‐to‐severe asthmatics were used for the REFS analysis.

Patients were not eligible for the study if they were pregnant, under the age of 18 or recently on omalizumab. mRNA expression was measured using an Illumina HumanHT‐12 V4.0 beadchip platform containing genes for approximately 20,000 transcripts.

### 2.2 Definition of disease severity and responders/non‐responders

Upchurch et al. (2020)^6^ selected asthmatic patients who had uncontrolled asthma despite treatment with inhaled corticosteroids (ICS) and/or long‐acting *β*‐agonists (LABA). Patients and non‐asthmatic healthy controls^6^ were recruited under protocols approved by the Institutional Review Board.

According to the Global Initiative for Asthma guideline,^27^ moderate asthma patients are well‐controlled with low or medium dose ICS and/or LABA, and severe asthma patients were defined as those that remain uncontrolled despite optimized treatment with high dose ICS‐LABA, or that require high dose ICS‐LABA to prevent asthma from becoming uncontrolled.

Disease severity was based on a combination of asthma control test (ACT), low lung function (FEV1 < 80%) and symptom frequency, including total number of days with symptoms per week and of night‐time awakenings per week.^6^

Omalizumab responsiveness was defined by Upchurch et al (2020)^6^ as improvements in asthma control, with non‐responders’ not able to achieve asthma control. Uncontrolled asthma was defined as a combination of factors including ACT score (<19), asthma‐related symptoms in number of days in previous week, use of short‐acting *β*‐agonists (≥2 × per week) and night‐time sleep disruption (≥2 per week), unchanged asthma control through medication, and indications of little/no improvements in asthma by physicians.^6^

### 2.3 Feature selection

To analyze the mRNA expression data, genes from the microarray platform Illumina were converted to gene IDs using the web probe conversion tool in the Ensembl Genome Browser.^28^ This step was done only with the final short list of genes. Next, the data were normalized with z‐score normalization.^29^

Non‐responders were labelled as 0 (*n* = 10), and responders were labelled as 1 (*n* = 30).

#### 2.3.1 Algorithm 1: REFS

REFS^30^ is an algorithm to discover features (genes) in our study. REFS was used to identify a gene signature in moderate‐to‐severe asthma patients to separate omalizumab responders from non‐responders. The REFS algorithm decreases the number of genes in the dataset to the most important genes associated with treatment outcome. REFS uses 8 different classifier algorithms: Gradient boosting, Passive Aggressive, Logistic Regression, Support Vector Machine classifier (SVC), Random Forest, Stochastic Gradient Descent, Ridge and Bagging. REFS merges the results of these individual classifiers. From these classifiers (metrics for algorithm performance), important genes are extracted, ranked and the lowest‐scoring genes are removed.

The REFS algorithm gives each gene associated with treatment outcome a ‘score’ based on how each different classifier algorithm uses it. For instance, in a tree‐based algorithm, the score is based on how many times a feature/gene was found in the tree. In the case of a coefficient‐based algorithm, the scoring depends on the coefficient value, with the highest weight being the most relevant.

As REFS is a stochastic algorithm, it was iterated 10 times. Running the algorithm several times with the same settings produces slightly different results and is thus a method to obtain more reliable results. After every iteration, the most relevant gene‐signature for therapy response is selected until the algorithm reaches 1 feature with a 20% step reduction and a first cut‐off of 1000 features.

The REFS algorithm was implemented on 28,402 genes and validated through 10‐fold cross‐validation.

To visualize the diagnostic ability of classifiers and evaluate the performance of learning models, Receiver Operating Characteristic (ROC) and area under the curve (AUC) were used.

### 2.4 Logic explained networks

While REFS is designed to directly perform feature selection for classification, the idea behind LENs is to automatically extract a set of rules that use a minimal number of features. In other words, REFS sees feature selection and classification as separate, while LEN attempts to perform both classification and feature selection in a single step. LEN was used to create a set of rules for predicting responsiveness to omalizumab on the same GSE134544 dataset.

#### 2.4.1 Algorithm 2: LEN

The LENs algorithm^31^ was implemented on 28,402 genes. LENs provide rules on sets of mRNA expression compared with healthy controls. It uses healthy controls as the baseline.

The LENs algorithm uses logic to transform gene expression values into qualitative descriptors that can be evaluated using a set of rules. LENs are a special family of concept‐based neural networks providing first‐order logic explanations for their decisions.^31^ Concept‐based models provide reasoning as in human‐interpretable symbols (the *concepts*). As an example, a concept‐based explanation to describe the category (*human*) can be through its characteristics (*head AND hand*), which leads to the explanation ‘a human has *hands* and a *head*’.

Logic‐based explanations may be merged to describe groups of observations. For instance, in an image dataset of humans, an image showing the anterior side of the face, a possible explanation to describe the category (*human*) can be through the attributes (*nose* AND *lips*), which reads ‘being *human* implies having a *nose* AND *lips*. Similarly, another image showing the posterior side of the *human* can be described through the attributes (*feet* AND *hair* AND *ears*). An explanation for the class *human* is then ‘being *human* implies having *feet* AND *hair* AND *ears*. Both explanations can then be merged using the attributes ‘(*nose* AND *lips*) OR (*feet* AND *hair* AND *ears*)’. The merged explanation reads then as ‘being *human* implies having a ‘(*nose* AND *lips)* OR (*feet* AND *hair* AND *ears)*’.

The quality of logic‐based explanations can be quantitatively measured to check their correctness and completeness (measurability). For instance, once the explanation “(*nose* AND *lips*) OR (*feet* AND *hair* AND *ears*)” is extracted for the class *human*, this logic formula can be applied to a test set to check its generality in terms of quantitative metrics like accuracy, and consistency.

In our research we compare responders and non‐responders and data from healthy controls used from LENs. LENs algorithm predicts a connected network of genes and their shared up/down regulated gene expression in omalizumab treatment comparing responders and non‐responders to mean mRNA expression of healthy controls. In this work, we used an Entropy‐based LEN^32^ implemented in the python package pytorch_explain.^33^

### 2.5 Biological context of gene signatures

To determine the biological context of the identified predictive genes for omalizumab therapy response and explain the (un)responsiveness to omalizumab, literature research was conducted. PubMed and Google Scholar were used for the assessment of gene function of each gene identified from REFS (Section 2.3.1) and LENs (Section 2.4.1). Protein function was investigated in the context of asthma pathology and omalizumab response.

## 3 RESULTS

### 3.1 Feature selection

The GSE134544 dataset containing 40 samples was used to run the REFS algorithm ten times (see Figure 3). With the machine‐learning REFS approach (Section 2.3.1) the total number of genes was reduced from 28,402 to 5 genes associated with therapeutic responsiveness. The optimal selection of genes depends on the classification accuracy of the binary problem (responders/non‐responders). The 5‐gene signature corresponds to the highest peak in accuracy over all 8 classifiers in the REFS ensemble (See, Figure S1). The gene expression values of responders, non‐responders and non‐asthmatic controls are shown in Figure 2.

### 3.2 Genes of interest

**FIGURE 1:**
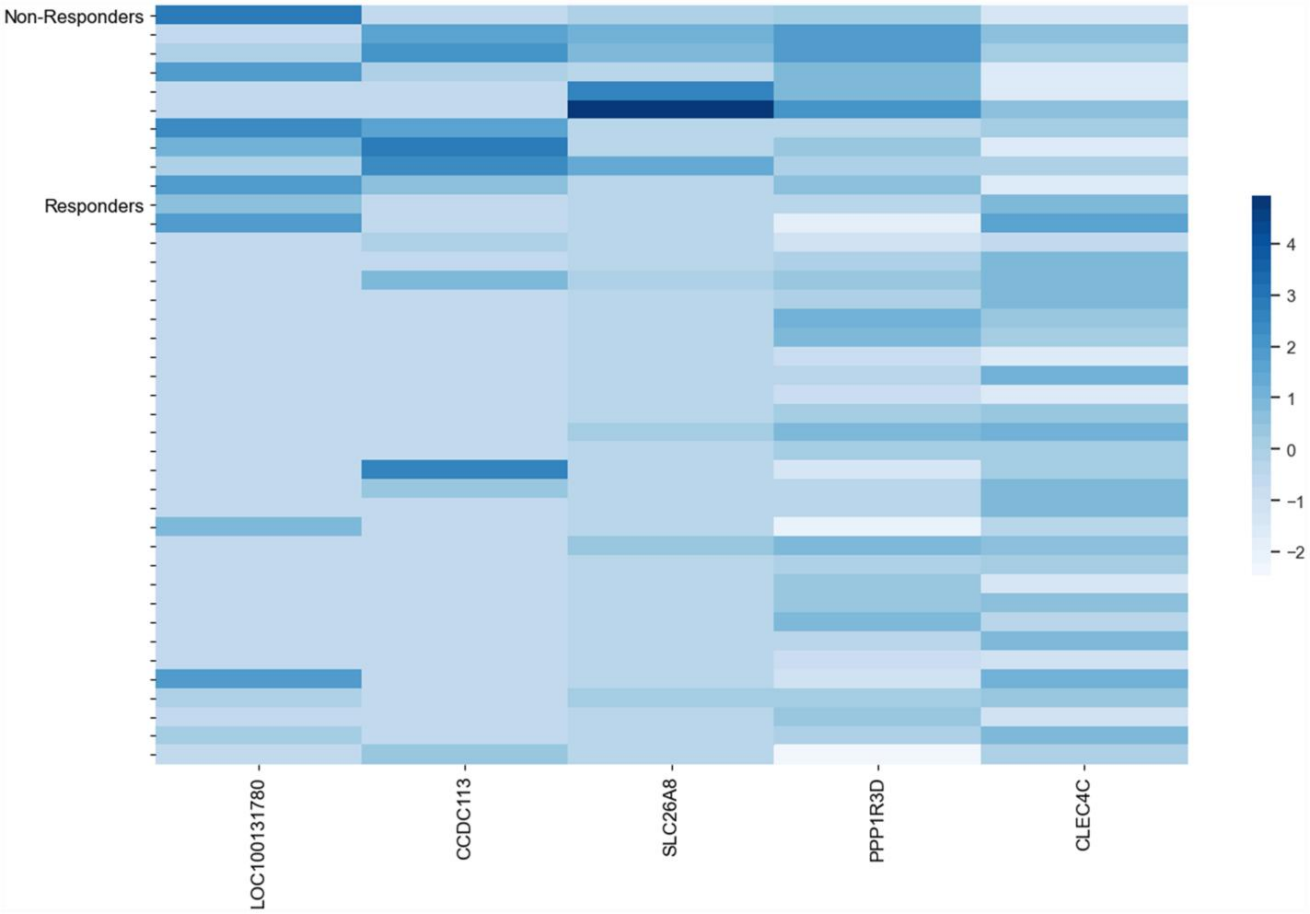
Heatmap of normalized gene expressions for the 5 selected genes in all samples identified by the Recursive Ensemble Feature Selection (REFS) algorithm (Section 2.3.1). The heatmap legend displays a color gradient range where −2 denotes the lowest gene expression and 4 the highest gene expression. Merely from visual inspection, samples can be differentiated into two groups: responders and non‐responders to omalizumab treatment.

REFS (Section 2.3.1) identified 5 individual genes as predictors of omalizumab response: *CCDC113, SLC26A, PPP1R3D, CLEC4C and LOC100131780*. The mRNA expression levels of the identified 5 genes in non‐responder patients' samples compared to responders are shown in Figure 1. Figure 1 shows that moderate‐to‐severe asthma patients who did not respond well to omalizumab have a higher mRNA expression of 4 out of 5 identified genes when compared to responders: *LOC100131780, CCDC113, SLC26A and PPP1R3D*. The mRNA expression of one gene, *CLEC4C*, is downregulated in non‐responders compared to responders.

### 3.3 ROC analysis of gene signature

**FIGURE 2:**
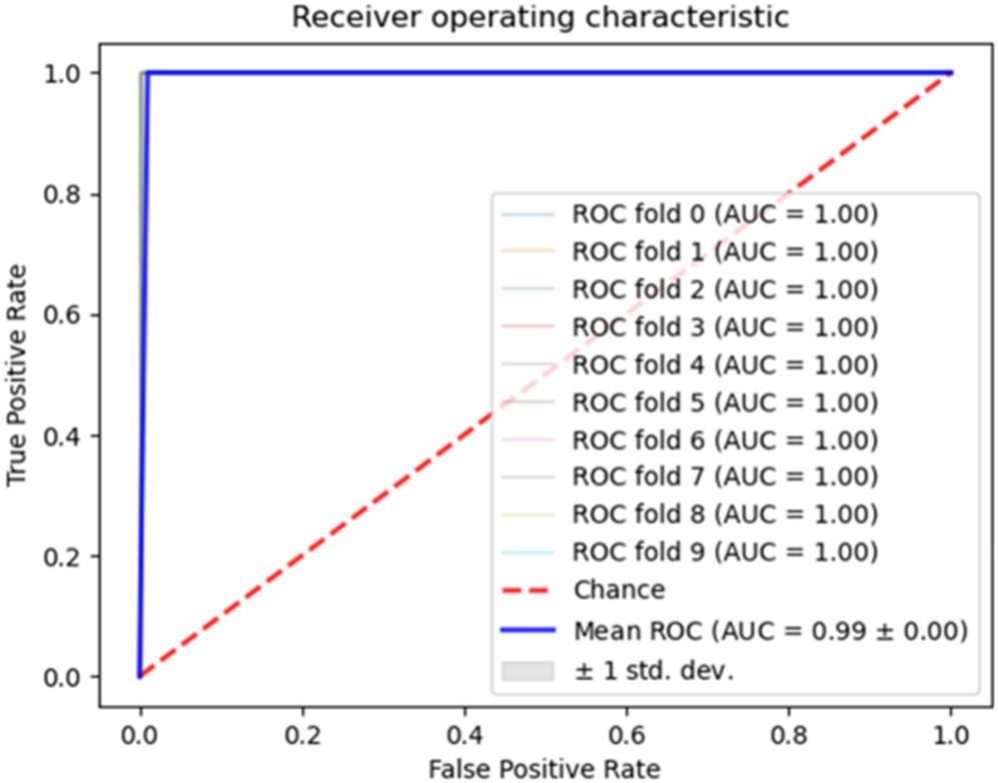
Receiver Operating Characteristic (ROC) curve over all 8 classifiers from Recursive Ensemble Feature Selection (REFS) to validate the identified 5‐gene signature. The ROC curve shows the binarization threshold from 0 (all moderate‐to‐severe asthma patients as omalizumab responders and both the TPR and FPR = 1; upper right corner of ROC) to 1 (all moderate‐severe asthma patients classified as non‐responders to omalizumab and both TPR and FPR = 0; lower left corner of ROC). The area under the curve (AUC) is the area in the plot which stays under the ROC curve. The Passive aggressive classifier which produced the blue ROC curve shows the best predictive accuracy as it covers a larger area compared to the straight ROC curve with the random classifier (red dashed line).

The AUC of the ROC curve was used to verify the efficacy/performance of the identified 5‐gene signature over all 8 classifiers in 10‐fold cross validation. As shown in Figure 2, the AUC of 0.99 was obtained using the Passive Aggressive classifier, a performance metric, which represents the best discriminatory accuracy for our model.^34^, ^35^

### 3.4 Relationship with treatment outcome: LEN

**FIGURE 3:**
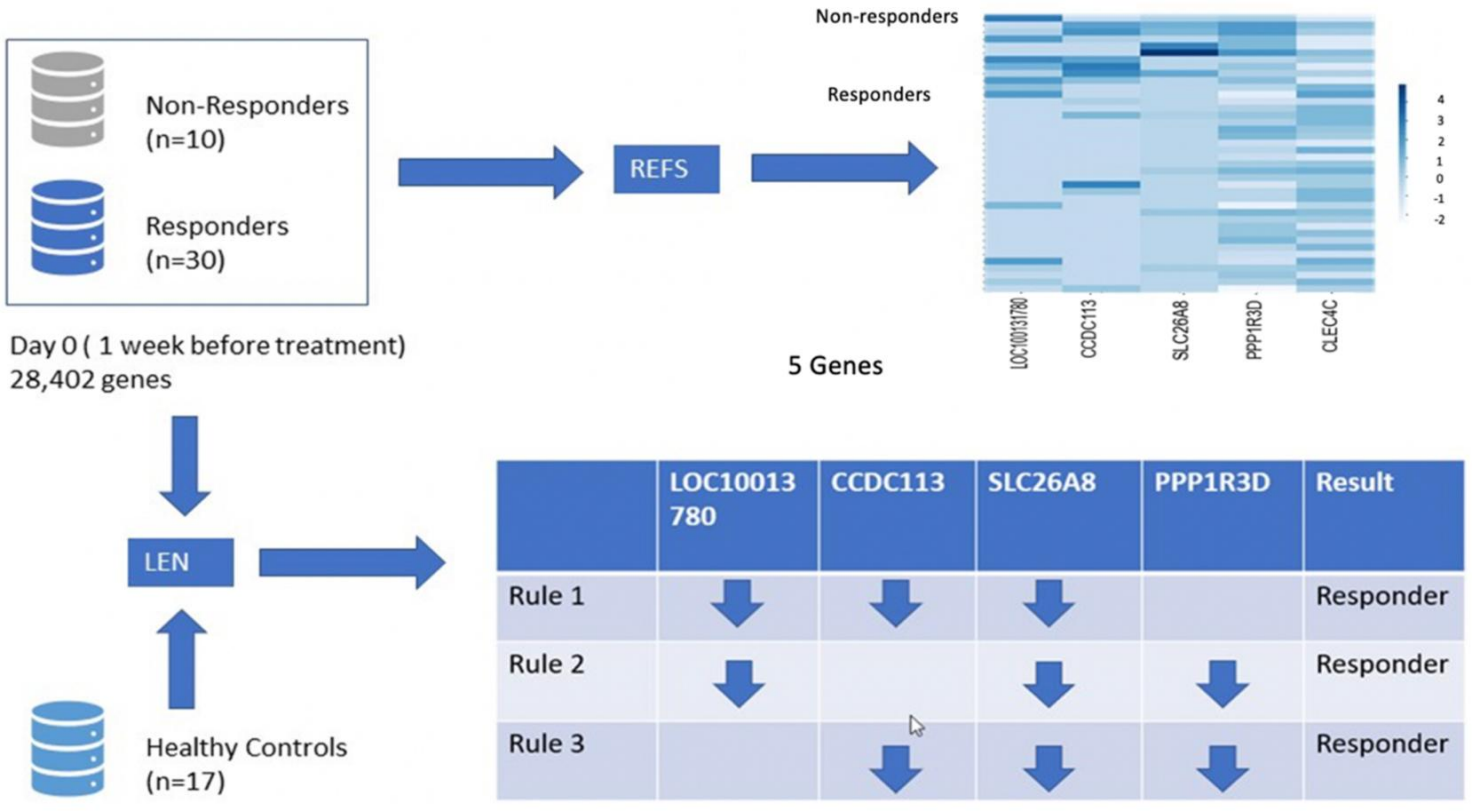
Summarized results of the two algorithms predicting treatment responsiveness of omalizumab in moderate‐to‐severe asthma. Whole blood mRNA expression profiles in samples collected day 0 (1 week before the start of the treatment) were used for the Recursive Ensemble Feature Selection (REFS) analysis (Section 2.3.1). In total, 40 moderate‐to‐severe asthmatic patients, *n* = 30 responders, and *n* = 10 non‐responders were included. For LEN analysis (Section 2.4.1), *n* = 17 healthy controls were also included. With REFS, five independent responsiveness‐predictive genes were identified, whereas rule‐based LEN identified three gene groups that predicted responsiveness. Comparing both approaches, an overlap of four genes was found. The relationship between responder status (R/NR) is shown in the heatmap. The mRNA expression of responders compared with healthy controls is shown in the table.

In parallel to the REFS algorithm (Section 2.3.1), a LEN on the same GSE134544 dataset was used to compare results (See, Figure 3).

As can be seen from Figure 3, the LEN algorithm returns three simple rules to explain why identified genes from the algorithm predict responsiveness to omalizumab.

The LEN rules 1, 2 or 3 predict the best responsiveness of omalizumab in moderate‐to‐severe asthma patients.

Rule 1 [LOC100131780, CCDC113 and SLC26A8] indicates a combination of response‐predictive gene set 1. The first rule implies that the mRNA expression of all genes in this trio is downregulated in the responder group as compared to the average mRNA expression of non‐asthmatic healthy controls.

Rule 2 [LOC100131780, SLC26A8 and PPP1R3D] indicates a combination of response‐predictive gene set 2. The second rule implies that the mRNA expression of all genes in this trio is downregulated in the responder group as compared to the average gene expression of non‐asthmatic healthy controls.

Rule 3 [CCDC113, SLC26A8 and PPP1R3D] indicates a combination of response‐predictive gene set 3. The third rule implies that the mRNA expression of all genes in this trio is downregulated in the responder group as compared to the average gene expression of non‐asthmatic healthy controls.

### 3.5 Combined results of REFS and LEN

The REFS (Section 2.3.1) and the rule‐based LEN (Section 2.4.1) both allow identification of compact gene signatures and provide a mean accuracy of 97.5%. An overlap in the results from both algorithms was found for 4 of the 5 genes identified by REFS. The following genes are identified as omalizumab response‐predictive genes: *LOC100131780, CCDC113, SLC26A* and *PPP1R3D*, which are all upregulated in non‐responders and downregulated in responders. This is summarized in Figure 3.

### 3.6 Biological interpretation of the gene signature predicting responsiveness to omalizumab

To determine the biological context of our findings, literature research on the proteins encoded by the five identified genes was conducted. Literature research showed that the identified response‐predictive genes can be associated with Mucosal immunity, Cellular energy, Airway hypersensitivity and remodeling. Details of the genes are enlisted in Table 1 and described below.

**TABLE 1:** Restricted set of features to predict responsiveness of Omalizumab (anti‐IgE therapy).

Publication	Gene	Protein	Cell	Functional context	Key findings
Chairakaki et al., 2018	*CLEC4C*	C‐type lectin domain family 4 member C	pDC	Mucosal and anti‐viral immunity (src pathway)	Implicated in driving acute asthma exacerbations Tolerogenic effect in asthma by inducing Treg cell differentiation
Vroman et al., 2017					
Firat‐Karalar et al., 2014	*CCDC113*	Coiled‐coil domain‐containing protein 113	Epithelial cells	Mucociliary clearance	Vital for ciliogenesis Reduction in cilium formation in knockdown models
Thomas, B et al., 2010					
Dirami, T et al., 2013	*SLC26A8*	Solute carrier family 26 member 8	Sperm cells	Male fertility	Associated with sperm motility Mutations may cause male infertility
Watson, C et al., 2017	*PPP1R3D*	Protein phosphatase 1 regulatory subunit 3D	Blood cells	Cell metabolism (AMPK pathway)	Regulates protein serine/threonine phosphatase activity Driver for acute peanut allergic responses
Zariwala, M. A et al., 2006	*LOC100131780*	Non‐annotated	Epithelial cells	Possible: Mucociliary clearance and airway remodelling	Overlaps partially with gene DNAI1 May be linked to primary ciliary dyskinesia
Lucas, J.S et al., 2014					

*Note*: Src family kinases (SFKs) are non‐receptor tyrosine kinases signaling coordinated cell proliferation, differentiation, apoptosis, migration, and metabolism.^36^, ^37^ 5‐adenosine monophosphate (AMP)‐activated protein kinase (AMPK) signalling pathway coordinates cell growth, autophagy and metabolism.^38^.

*CLEC4C*, also denoted as the *BDCA2* or *CD303* gene, encodes a member of the C‐type Lectin superfamily, C‐type lectin domain family 4 member C. This Lectin‐type cell surface receptor is linked to various functions including antigen‐uptake by dendritic cells^39^ for internalization/presentation to T cells^40^ and attachment to serum IgG.^41^ *CLEC4C* triggers the src‐family protein‐ tyrosine kinases signaling pathways to inhibit the induction of IFN‐α/β expression in plasmacytoid dendritic cells (pDC).^30^, ^39^ This inhibition, in turn, leads to the production of pro‐ or anti‐inflammatory cytokines and consequently, fine‐tunes innate and adaptive immune responses to viral infections. High mRNA expression of *CLEC4C* in whole blood has been associated with low mRNA expression of Toll‐like receptor 7 (TLR 7) expression and increased risks for common colds in asthmatic subjects. TLR‐7‐mediated induction of IFN‐ α/β, and inflammatory cytokine production is critical in antiviral immune responses.^42^ Hence, impaired anti‐viral response in asthmatic patients may be coordinated by *CLEC4C*.^43^ (See, Supporting Information S1) Taken together, the present set of findings indicate that severe asthma patients suffering from frequent (viral) exacerbations may have enhanced pDC (*CLEC4C* + DC) expression. This conclusion seems sensible as pDCs are critical mediators for anti‐viral responses. Our results obtained from the REFS algorithm show that *CLEC4C* expression in whole blood of non‐responders to omalizumab was downregulated when compared to responders. Thus, for a proper response to omalizumab, pDC may be central in improving asthma symptoms.

*CCDC113* gene encodes Coiled‐coil domain‐containing protein 113 *(CCDC113)*, which is a centrosome‐associated protein critical for cilia formation.^44^ This gene coordinates ciliary beating frequency, beat direction and cilia stroke.^45^ *CCDC113* has been shown to be overexpressed in the nasal brushes of asthmatic subjects when compared to healthy controls.^46^ Upregulation of *CCDC113* could possibly be a rebound effect for ciliary defects or damage. The upregulation of *CCDC113* shown in our results from LENs and REF can be expected based on the pathology of asthma. In the airways, cilia work along with airway mucus to facilitate mucociliary clearance acting as a pulmonary defense.^47^, ^48^ Therefore, it follows that ciliary dysfunction could lead to impaired mucociliary clearance, making asthmatic patients more susceptible to airway infection or inflammation. Overexpression of *CCDC113* mRNA in the whole blood of non‐responders compared with omalizumab responders might indicate ciliary defects in the airways of non‐responders. Thus, as *CCDC113* is involved in the cilium assembly, it may simply increase the severity of asthma.

The *SLC26A8* gene encodes Testis anion transporter 1, which is a sperm‐specific member of the SLC26 family of multifunctional anion exchangers. *SLC26A8* functional relevance is linked to sperm motility and mutations appear to cause male infertility.^49^ In the whole blood mRNA of severe asthmatics, upregulated *SLC26A8* compared to healthy controls has been reported.^50^ The presented LENs and REFS approach showed similar results for non‐responders. Increased levels of *SLC26A8* are associated with asthma and lower sperm count/sperm motility.^51^ Given the limited information, it is hypothesized that *SLC26A8* is merely an additional biomarker in non‐responders as no logical context related to omalizumab therapy response can be formulated.

*PPP1R3D*, encodes for Protein Phosphatase 1 (PP1) regulatory Subunit 3D.^52^ This gene is associated with the cellular energy sensor 5′ AMP‐activated protein kinase (AMPK).^52^ The interaction between AMPK and PP1 is regulated by intracellular glycogen content.^51^ Upregulated mRNA expression of *PPP1R3D* in blood cells is associated with IgE‐mediated peanut allergies in children.^53^ Food allergies are also associated with increased asthma severity in later life.^54^, ^55^, ^56^ Interestingly, *PPP1R3D* has been reported as a novel obesity candidate gene.^57^ Obese adults tend to have more severe asthma compared to lean adults.^58^ Indeed, *PPP1R3D* was found to be overexpressed by LENs and REFS in the non‐responder group. Based on the limited information on this gene, it can be suggested that non‐responders to omalizumab might exhibit a severe non‐IgE‐mediated form of asthma or might be more obese than responders. Data on body weight of asthmatic subjects were not available for inclusion in the present study.

*LOC100131780* is an illumina gene ID, which could not be matched to a specific gene in the literature. As such, the association of *LOC100131780* with asthma remains obscure. Limited information could be recovered computationally from its sequence overlap with *DNAI1* that codes for dynein axonemal intermediate chain 1. *LOC100131780* as *CCDC113* also points to the important role of primary cilia. *Mutations of the LOC100131780* gene^59^ have been linked to primary ciliary dyskinesia (PCD), a rare inherited disease with dysfunctional mucocilIary clearance eventually leading to airway remodeling in thickened airway walls and obstruction.^60^, ^61^ Elevated levels of *LOC100131780* were found by LENs and REFS in omalizumab non‐responders. Speculatively, PCD is a confounding condition that mimics asthma symptoms. As PCD and asthma rarely co‐exist^60^ patients with overexpressed *LOC100131780* classified as non‐responders might be misdiagnosed with severe asthma instead of PCD.

## 4 DISCUSSION

Pre‐therapeutic screening helps in unwarranted drug exposure in patients suffering from severe forms of asthma. Treatment with a biological agent is the most logical step for this group of patients. However, different biologicals are available and biomarkers to determine which biological would be appropriate for the individual patient are lacking. Omaluzimab (anti‐IgE) is one of the commonly used biologicals in asthma patients; however, patients do not respond equally well to therapy with this drug.^21^, ^62^, ^63^ Studies examining transcriptional expression profiles in whole blood and sputum of asthmatic patients have identified gene signatures that may be associated with asthmatic phenotypes^64^, ^65^, ^66^ or therapeutic responsiveness to ICS^67^, ^68^ and omalizumab.^6^, ^18^ However, no definitive set of biomarkers for omalizumab therapy response has been found till date, while omalizumab response prediction could be very beneficial for proper disease management. Therefore, better predictive biomarkers for omalizumab treatment response are needed to select the best treatment strategy for severe asthma patients.

ML prediction can help to find a small and easy to measure a set of biomarkers that predicts treatment responses. However, finding accurate and robust response‐predictive genes using ML has proven to be a challenge due to underpowered studies, poor explanatory models and the use of single genes or gene sets with no or few overlapping genes.^22^, ^23^ REFS algorithm offers a solution for this issue^25^, ^69^ in terms of better accuracy and robustness. LEN can explain why the algorithm arrives at a certain decision, such that the results of the LEN algorithm can be interpreted by clinicians. Explainability matters especially in a clinical context because predictions are useful when they can be understood for the acceptance of the AI decisions by physicians. LENs^26^, ^31^, ^32^ is an interesting approach to extract biologically meaningful gene associations and thereby increase prediction model interpretability.

In the present study, a novel feature selection algorithm combining 8 classifiers, REFS, was used on open accessible mRNA expression data (GSE13544) from moderate‐to‐severe asthma patients to predict treatment responsiveness of omalizumab. In addition to REFS, the entropy‐based LEN model^26^ was used, as it provides the highest flexibility, generalization accuracy, and less complex logic formulas. The machine‐learning‐based REFS algorithm returned a 5‐gene signature: *CLEC4C, CCDC113, SLC26A8, PPP1R3D* and *LOC100131780*. The LEN algorithm returned a short 4‐gene signature: *CCDC113, SLC26A8, PPP1R3D* and *LOC100131780*. Groups of responsive predictive genes: ([*LOC100131780, SLC26A8* and *PPP1R3D*]; [*LOC100131780, CCDC113* and *SLC26A8*]; [*CCDC113, SLC26A8* and *PPP1R3D*]) were found through the LEN algorithm. Four overlapping genes (*CCDC113, SLC26A8, PPP1R3D* and *LOC100131780*) were found to be upregulated in non‐responders with both methodologies. The gene expression values of 5 selected genes across healthy controls, non‐responders, and responders are shown in Figure S2. Our compact set of genes was able to differentiate between responders and non‐responders with a mean accuracy 0.975. As shown in Figure S2, after treatment with omalizumab, the gene expression levels of the 5 identified genes of responders were closer to the healthy controls.

Zhang et al (2021),^18^ used the same GEO dataset GSE134544 employed in this study. The study reported one useful genetic biomarker, the T‐cell surface glycoprotein CD3 epsilon chain (CD3E) using weighted gene co‐expression network analysis. This biomarker was downregulated in non‐responders. However, a single biomarker might be insufficient to capture interactions between other key genes relevant to accurately predict patient responsiveness to omalizumab treatment. For example, some patients exhibiting high CD3E expression might still not respond to omalizumab, whereas conversely, some patients with low CD3E expression could respond to treatment. Identifying a short list of responsiveness predictive genes as in our results could be useful in clinical settings to show coordinated changes in gene expression that may impact the therapeutic response, which cannot be identified by a single biomarker. With REFS/LENs method a mean accuracy of 0.975 was computed and the proposed methodology outperformed Zhang et al. (2021)^18^ that reported an accuracy of 0.763. The utility and accuracy of REFS in comparison to other methodologies has been previously demonstrated in miRNA and mRNA datasets.^24^, ^25^

Upchurch et al. (2020)^6^ identified biomarkers through gene clustering but predicted a large set of response‐predictive genes on GSE134544. With the REFS and LEN approach, a more compact set of genes (4) in contrast to Upchurch et al. (2020)^6^ methodology (1776) was identified. Overall, the results from REFS and LENs show robustness and higher accuracy.

To validate the relevance of overlapping genes from both computational methodologies and understand the mechanisms involved in omalizumab responsiveness, biological functions of the genes were investigated. From a clinical perspective, every single gene in the predicted gene set by REFS is a potential biomarker for omalizumab therapy response. How large the contribution of genes in the predicted gene trios identified by LENs is, is a matter of debate. As the mRNA of all genes are overexpressed in the non‐responder group, it can mean that these combinations are of interest for further research due to their supposed increased biological significance in activating or overloading specific biological pathways related to asthma pathogenesis and omalizumab response.

Co‐expression of response‐predictive genes does not simply mean interaction between their associated proteins, but the results may propose similarities in their regulation by transcription factors such as NFκb which controls various aspects of innate and adaptive immune functions and serves as a critical mediator of inflammatory responses.^70^ In the current study, genes that were differentially co‐expressed between responders and non‐responders were associated in pathways regulating diverse pathological processes in chronic inflammatory conditions (the AMPK pathway *PPP1R3D* or tyrosine kinase signaling pathway (*CLEC4C*) associated with disturbed mucosal clearance (*CCDC113* & *LOC100131780*) and tissue remodeling (*LOC100131780*) (See, Table 1). These genes appear to be somewhat connected in terms of function. It is possible that the upregulated genes in non‐responders are associated with a more severe asthmatic phenotype. That might be a reason why patients with severe asthma might respond badly to omalizumab.

The severity of asthma could result from impaired immune activation or dysregulation of the immune response. Zhang et al (2021),^18^ hypothesized that omalizumab inhibits airway inflammation by reducing the Th2 inflammation cascade. By calculating the immune enrichment score, they found that CD4+ T and dendritic cell numbers were lower in the blood of non‐responders. A low T‐cell signature and high inflammatory gene cluster in non‐responders to omalizumab was also reported by Upchurch et al. (2020).^6^ In the patient cohort, it is possible that type 2 inflammation indicative for IgE‐mediated asthma and non‐type 2 inflammation might not be entirely mutually exclusive in responders and non‐responders as found by Zhang et al (2021)^18^ or Upchurch et al. (2020).^6^ Although we did not investigate this aspect in our study, mixed phenotypes (type 2 and non‐type 2) might be associated in the same individual, resulting in less likelihood of response to omalizumab.

One of the most intriguing results was the identification *SLC26A8* linked to male infertility as an mRNA predictor in all LEN proposed gene combinations. Although not much is known about this biological function of this gene in asthma, it might play a pivotal role in regulating gene expression changes observed in the airways of male non‐responders to omalizumab therapy (See, Table 1). Determining the interplay between asthma severity, gender and reproductive health was beyond the scope of this study.

Our findings thus reflect that predicting response to omalizumab therapy is powerful when identified genes are given a biological context. However, our approach does have some limitations. Unfortunately, we could not find an independent cohort dataset to validate our gene signature. Not all pathways involved in our gene set have been fully characterized in the literature. Consequently, our findings depend on the current state of knowledge and pathway annotations of genes may be sparse and skewed in comparison to well‐defined gene sets. To complement our literature search, in vitro experiments with whole blood samples might provide us with broader insight regarding gene function and mechanisms associated with response to omalizumab. For clinical applications, a simple blood test can be useful to assess the mRNA expression of the 4 overlapping genes for future treatment with omalizumab. If mRNA expressions of all 4 overlapping genes are found to be downregulated in the responder group compared with healthy control mRNA expression, anti‐IgE treatment might be beneficial for the patient.

In summary, we suggest that our small set of mRNA predictors returned from two different algorithms is highly accurate and holds potential value as clinical biomarkers for predicting omalizumab treatment response in moderate‐to‐severe asthma. We conclude that our work represents a first step towards a more tailored prediction of omalizumab response. Future studies in other cohorts should validate our computational approach. Furthermore, a prospective study is necessary to test the clinical utility of the biomarkers. As a next step, add‐on studies comparing biological medications could be valuable to identify patients who should be treated with omalizumab versus another biological. To support physicians' treatment decision for a biological, we recommend confirming the diagnosis of severe asthma, exclusion of conditions mimicking asthma symptoms such as PCD and assessment of comorbidities associated with severe forms of asthma. Patients who might attain incomplete benefit from omalizumab could benefit from switching to a different biologic which targets an alternative mechanism if GINA criteria are met. Alternatively, non‐responders might benefit from add‐on therapy with a second biologic.

## AUTHOR CONTRIBUTIONS

**Sarah Kidwai**: Conceptualization (equal); Investigation (equal); Writing – original draft (lead). **Pietro Barbiero**: Methodology (lead); Validation (lead); Writing – review & editing (equal). **Irma Meijerman**: Supervision (lead); Writing – original draft (equal); Writing – review & editing (equal). **Alberto Tonda**: Formal analysis (lead); Methodology (equal); Writing – review & editing (equal). **Paula Perez‐Pardo**: Resources (equal); Writing – original draft (equal); Writing – review & editing (equal). **Pietro Lio**: Supervision (equal); Writing – original draft (equal); Writing – review & editing (equal). **Anke H. Maitland‐van der Zee**: Conceptualization (equal); Writing – original draft (equal); Writing – review & editing (equal). **Daniel L. Oberski**: Supervision (lead); Writing – original draft (equal); Writing – review & editing (equal). **Aletta D. Kraneveld**: Conceptualization (lead); Supervision (lead); Writing – original draft (equal); Writing – review & editing (equal). **Alejandro Lopez‐Rincon**: Conceptualization (equal); Methodology (lead); Visualization (lead); Writing – review & editing (equal).

## CONFLICT OF INTEREST STATEMENT

All authors of the manuscript declare that the research was conducted in the absence of any commercial or financial relationships that could be construed as a potential conflict of interest.

## Supporting information

Supporting Information S1

## Data Availability

All the necessary scripts to reproduce the experiments are stored on the public GitHub repository: https://github.com/steppenwolf0/REFS. We used clinical and array data from the Gene Expression Omnibus (GEO) with accession code GSE134544.
