# Supplementary material for: A robust mRNA signature obtained via recursive ensemble feature selection predicts the responsiveness of omalizumab in moderate‐to‐severe asthma
Source: Clin Transl Allergy. 2023 Nov 17;13(11):e12306. doi: 10.1002/clt2.12306 (PMC10655633; doi:10.1002/clt2.12306)
Supplement: Supplementary file 1 — Supporting Information S1 [file CLT2-13-e12306-s001.docx]

Supplementary Materials for “**A robust mRNA signature obtained via Recursive Ensemble Feature Selection predicts the responsiveness of omalizumab in moderate-to-severe asthma**”

**S. Kidwai**1**, P. Barbiero**3**, I. Meijerman**1**, A. Tonda**4**, P.Perez-Pardo**1 **P. Lio ́** 3**, A. H. Maitland-van der Zee**5 **D. L. Oberski**2**, A.D Kraneveld**^1^ and **A. Lopez-Rincon** 1,2

1Division of Pharmacology,Utrecht Institute for Pharmaceutical Science, Faculty of Science,Utrecht University,Universiteitsweg 99, 3584 CG Utrecht,the Netherlands
2Department of Data Science, University Medical Center Utrecht
3Department of Computer Science and Technology, University of Cambridge, 15 JJ Thomson Avenue, Cambridge CB3 0FD, United Kindgdom
4UMR 518 MIA, INRAE, Universite Paris-Saclay, Paris, France
5Department of Pulmonary Medicine, Amsterdam UMC, University of Amsterdam, Amsterdam, Netherlands

Supplementary Figures


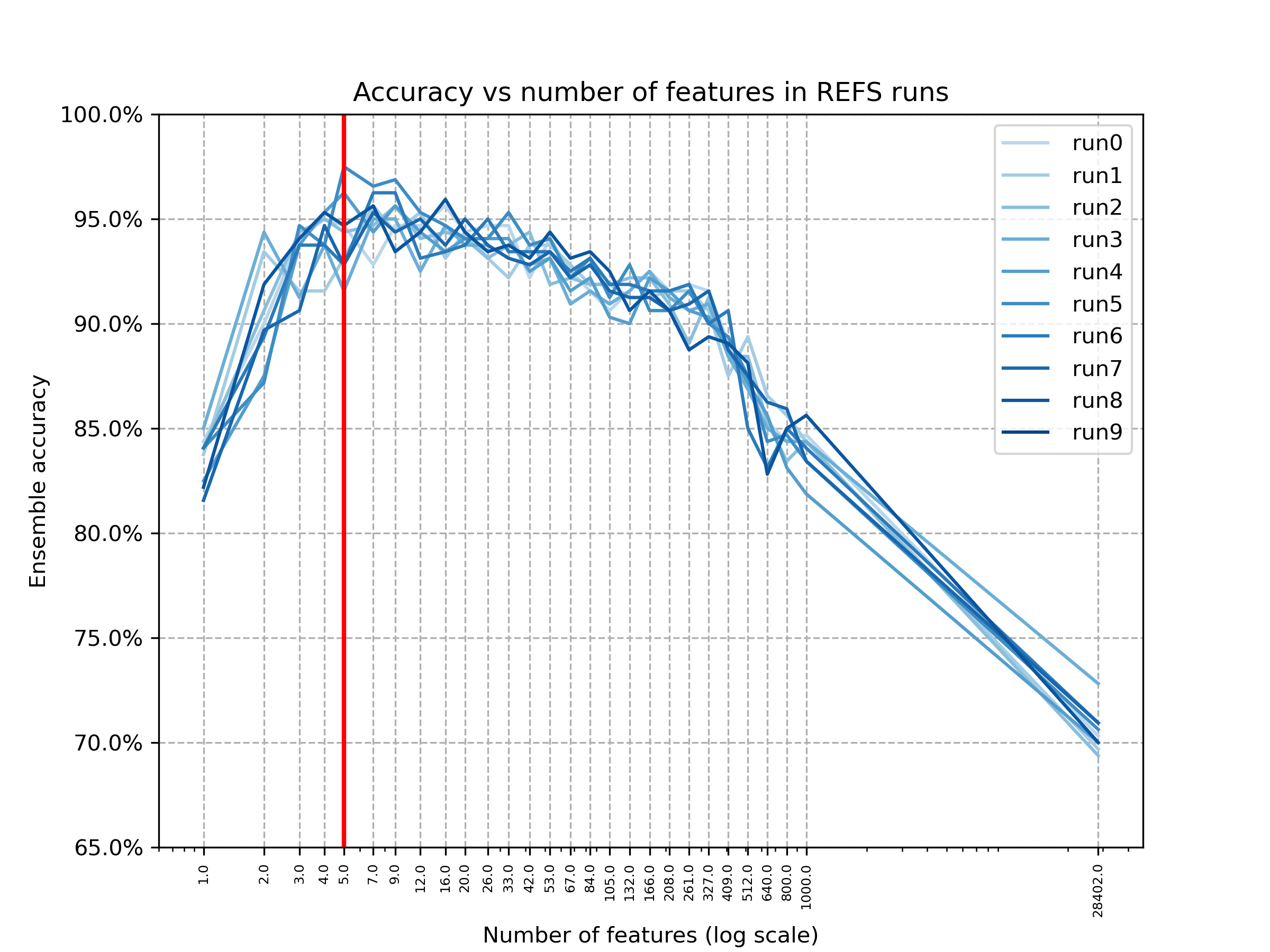


**Figure S1.**  **10 runs of the REFS algorithm** The REFS algorithm uses multiple classifiers and runs these 10 times for its decision process. The results of the REFS algorithm run in ten-fold for the classification of treatment response to omalizumab in moderate-to-severe asthma patients is shown here. The X axis cuts at 5 variables, suggesting that 5 genes are optimal for response prediction. The 5-gene signature corresponds with the highest peak in accuracy over all 8 classifiers in the REFS ensemble.

***
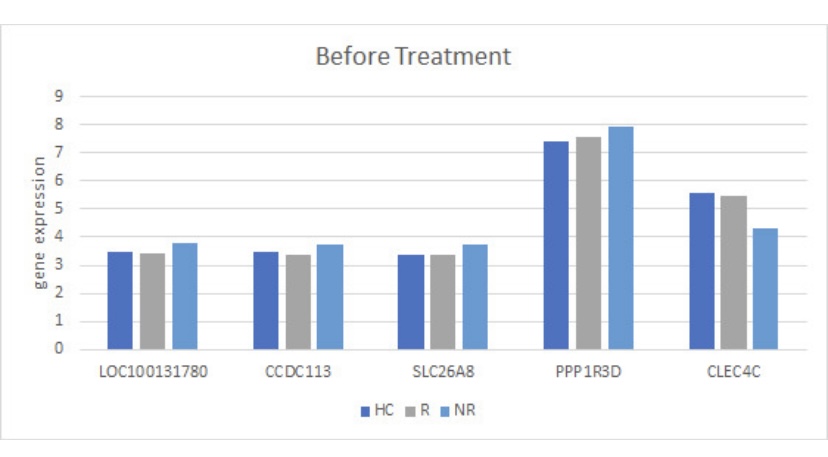
***

***
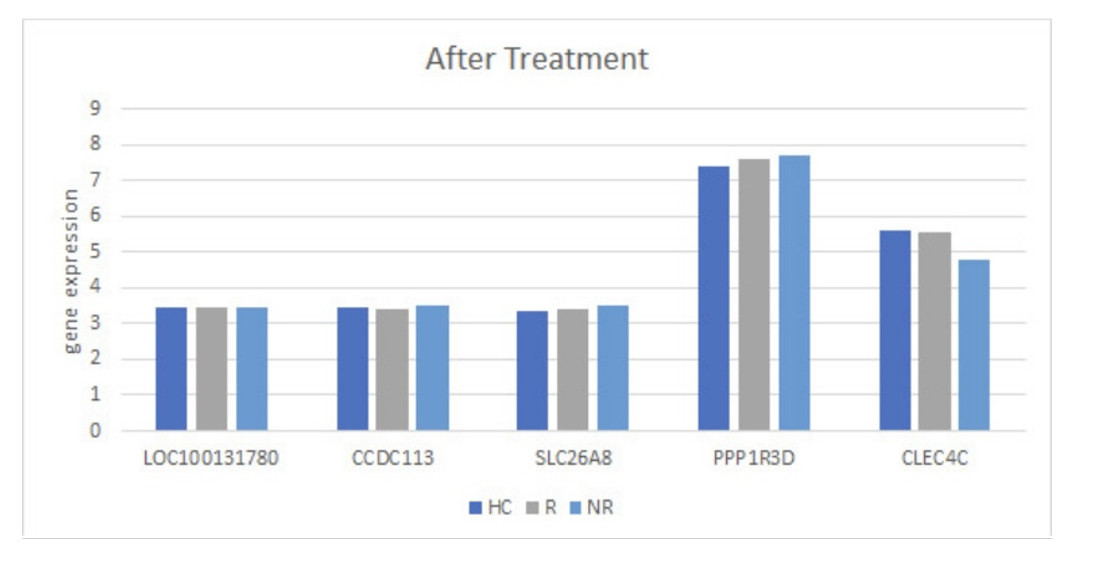
***

**Figure S2.** Genes expression values for comparison of 5 selected genes across healthy/responders/non-responders before (upper)and after treatment (lower) with Omalizumab.

Supplementary Text
S1 **Biological interpretation of the gene signature predicting responsiveness to omalizumab**

*CLEC4C:* Studies have shown that *CLEC4C* is a marker for pDCs,1-3 associated with increased asthma-related inflammation and exacerbations. 4,5 Lin et al (2021) 3 reported increased production of IFN-α in pDCs in healthy controls as compared to pDCs of asthmatic patients under TLR-7 stimulation and prolonged rhinovirus (RV) stimulation. These results suggest an impaired function of pDCs in asthma under prolonged RV stimulation, compromising TLR-7 stimulated production of IFN-α in pDC and virus clearance. Thus, under TLR-7 stimulation, the production IFN-α in pDCs is defective in asthmatic patients pointing towards an impaired innate response and subspeciality to RV infection in asthma.

Spears et al (2011) 6 showed that asthma patients exhibit a larger increase in peripheral blood DCs including CLEC4C+pDC. In murine models for asthma, 7 CLEC4C+pDC appeared to reduce airway hyperresponsiveness and inflammation through suppression of type 2 innate lymphoid cells (ILC2), thereby inhibiting secretion of pro-allergic cytokines. Furthermore, another study 4 showed that pDC depletion after allergen challenge or during RV infection may abrogate exacerbations and inflammation by IL-25 in murine models for allergic asthma. These findings suggest that virus-induced exacerbations are associated with an increased pDC infiltration into airways. Consistently, pDC numbers (*CLEC4C*+DC) in sputum of asthmatic patients were significantly enhanced during exacerbation and associated with increased inflammation severity and increased risk of exacerbations. 4

*SLC26A8:* As *SLC26A8* has been associated with male infertility, it might be of interest to explore gender differences in omalizumab therapy response. Increased levels of *SLC26A8* in males might indicate an interplay between asthma severity and reproductive health which can influence therapy response. However, retrospective analysis of anti-IgE therapy did not reveal significant differences between gender and therapeutic responsiveness. 8,9 Interestingly, *another member of the family, SLC26A6* has been associated with transporting chloride, oxalate, sulfate and bicarbonate. 10 Chloride transporters proteins play pivotal roles in inflammatory airway diseases, contributing to harmful effect of mucus overproduction, and airway obstruction. It is unknown if SLC26A8 contributes to innate immune properties of mucus and mucociliary clearance.

*PPP1R3D*: Genes encoding for other subunits of PP1 such as PPP1R16A in blood cells was found overexpressed in patients with asthma and PPP1R15A in alveolar cells is overexpressed in corticosteroid resistant asthmatic patients.11 Interestingly, *PPP1R3D* has been reported as a novel obesity candidate gene.12 Severe asthma phenotypes include type 2 (T2) indicative for IgE-mediated asthma and non-type 2 (not T2) asthma. 13 Studies have suggested that obese females have a low T2 signature and are less likely to respond to omalizumab treatment. 14

# References

1. Murray, L. M., Yerkovich, S. T., Ferreira, M. A. & Upham, J. W. Risks for cold frequency vary by sex: role of asthma, age, tlr7 and leukocyte subsets. *Eur. Respir. J.* 56 (2020).
2. Murray, L., Xi, Y. & Upham, J. W. Clec4c gene expression can be used to quantify circulating plasmacytoid dendritic cells. *J. immunological methods* 464, 126–130 (2019).
3. Lin, T.-Y. *et al.* Impaired interferon-α expression in plasmacytoid dendritic cells in asthma. *Immunity, inflammation disease* 9, 183–195 (2021).
4. Chairakaki, A.-D. *et al.* Plasmacytoid dendritic cells drive acute asthma exacerbations. *J. Allergy Clin. Immunol.* 142, 542–556 (2018).
5. Vroman,H.,Hendriks,R.W.&Kool,M.Dendriticcellsubsetsinasthma:impairedtoleranceorexaggeratedinflammation? *Front. immunology* 8, 941 (2017).
6. Spears, M. *et al.* Peripheral blood dendritic cell subtypes are significantly elevated in subjects with asthma. *Clin. & Exp. Allergy* 41, 665–672 (2011).
7. Maazi,H.*etal.*Activatedplasmacytoiddendriticcellsregulatetype2innatelymphoidcell–mediatedairwayhyperreactivity. *J. Allergy Clin. Immunol.* 141, 893–905 (2018).
8. Bousquet, J. *et al.* The effect of treatment with omalizumab, an anti-ige antibody, on asthma exacerbations and emergency medical visits in patients with severe persistent asthma. *Allergy* 60, 302–308 (2005).
9. Viswanathan, R. K., Moss, M. H. & Mathur, S. K. Retrospective analysis of the efficacy of omalizumab in chronic refractory urticaria. In *Allergy and asthma proceedings*, vol. 34, 446 (OceanSide Publications, 2013).
10. Sala-Rabanal, M., Yurtsever, Z., Berry, K. N. & Brett, T. J. Novel roles for chloride channels, exchangers, and regulators in chronic inflammatory airway diseases. Mediat. inflammation 2015 (2015).
11. Alrashoudi, R. H., Crane, I. J., Wilson, H. M., Al-Alwan, M. & Alajez, N. M. Gene expression data analysis identifies multiple deregulated pathways in patients with asthma. *Biosci. Reports* 38, DOI: 10.1042/BSR20180548 (2018).
12. Morton, N. M. *et al.* A stratified transcriptomics analysis of polygenic fat and lean mouse adipose tissues identifies novel candidate obesity genes. *PloS one* 6, e23944 (2011).
13. De Ferrari, L. *et al.* Molecular phenotyping and biomarker development: Are we on our way towards targeted therapy for severe asthma? *Expert. Rev. Respir. Medicine* 10, 29–38, DOI: 10.1586/17476348.2016.1111763 (2016).
14. Kuruvilla, M. E., Lee, F. E. H. & Lee, G. B. Understanding Asthma Phenotypes, Endotypes, and Mechanisms of Disease. *Clin. Rev. Allergy Immunol.* 56, 219–233, DOI: 10.1007/s12016-018-8712-1 (2019).
